# Predicting immune checkpoint inhibitors response via fluorescence lifetime imaging microscopy: a systematic review

**DOI:** 10.3389/fimmu.2025.1626608

**Published:** 2025-10-06

**Authors:** Carlo Cossa, Giulio Frigato, Massimo Lupo, Gabriele Mazzeo, Alex Moro, Francesco Patrucco, Reanna Wang, Andrea Doni, Piergiuseppe Colombo

**Affiliations:** ^1^ Humanitas University, Milan, Italy; ^2^ Unit of Multiscale and Nanostructural Imaging, IRCCS Humanitas Research Hospital, Milano, Italy; ^3^ Department of Biomedical Sciences, Humanitas University, Milan, Italy; ^4^ Pathology Department, IRCCS Humanitas Research Hospital, Milan, Italy

**Keywords:** FLIM, ICI, IFRET, PD1, PDL1, CTLA4, immunotherapy

## Abstract

**Introduction:**

Fluorescence Lifetime Imaging Microscopy (FLIM) is an imaging technique that allows for the visualization of the cellular microenvironment by measuring the decay time of endogenous fluorescent molecules. Its advent has allowed the acquisition of information on previously undetectable aspects of the tissue environment, which also includes some mechanisms involving immune checkpoints. Understanding the level of interaction with their ligands is of paramount importance when stratifying patients for immunotherapy, as traditional methods such as immunohistochemistry (IHC) were found to be ineffective in predicting responders.

**Methods:**

This review analyzes the current literature on FLIM as a means of predicting targets’ responsiveness to ICIs by examining the most relevant databases. Following PRISMA guidelines, we identified the relevant literature. The predefined objective of this review was to evaluate the potential of FLIM as a predictive biomarker of responsiveness to immune checkpoint inhibitors (ICIs). Eligibility criteria included original studies (clinical or preclinical) reporting on the use of FLIM to assess tumor or immune microenvironment in the context of ICI therapy. Reviews, case reports, editorials, and abstracts without full text were excluded.

**Results:**

Research suggests that interaction, not expression, is positively correlated with the effectiveness of ICI treatment. FLIM, in combination with FRET, allows for the quantification of the interactions within the tumor microenvironment.

**Discussion:**

The scope of the review is to assist researchers in further exploring this technology for possible applications and for future drug interaction studies.

## 1 Introduction

The advent of Immune Checkpoint Inhibitors (ICI) revolutionized oncological therapies by enabling the immune system to fight against cancer. Despite their effectiveness on many tumors, only 20-40% (1) of patients are estimated to respond to immunotherapy. One of the main issues is represented by non-responders. Given the high cost of treatment and potential side effects, developing reliable methods for predicting patients’ response to these drugs is paramount.

### 1.1 The issue

The predictive value of ICI response is traditionally based on the evaluation of immunohistochemical (IHC) expression of specific proteins (i.e., PD-L1) detectable in patients’ neoplastic tissue, mostly in the advanced stages of the disease. The PD-1/PD-L1 and CTLA-4 axes are described in detail in the **Supplementary Material**. In the last 15 years, advanced automated techniques have been developed for the preparation of stained sections with monoclonal antibodies to minimize interpretation errors and standardize immunohistochemical analysis. However, this method is not without limitations, leading to very low predictive value and poor patient stratification. It has been demonstrated that some patients with high PD-L1 expression do not respond to ICIs. In contrast, others with low or absent PD-L1 expression may still derive benefit, highlighting the imperfect nature of this biomarker (2).

In the pursuit of more accurate and reliable methods for visualizing molecular interactions, researchers have turned to advanced imaging techniques like Fluorescence Lifetime Imaging Microscopy (FLIM) and Förster Resonance Energy Transfer (FRET). FLIM, which emerged in the late 1980s, initially focused on measuring the decay rates of fluorescent molecules, offering a novel way to study the microenvironment of these molecules beyond what was possible with traditional fluorescence intensity imaging. Over the decades, continuous technological advancements and refinements have expanded FLIM’s applications, making it a crucial tool for real-time visualization of molecular dynamics. Today, FLIM, often coupled with FRET, allows for the detailed examination of the molecular environment and interactions through autofluorescence of cellular components (e.g., NADH, collagen) or fluorescent probes labeling target molecules. These technologies provide high spatial and temporal resolution (3), enabling researchers to gain deep insights into cellular processes and molecular interactions, thus paving the way for breakthroughs in fields like cancer research and immunotherapy.

Current research has focused primarily on PD-1 and its ligand PD-L1, but new research is emerging on CTLA-4. With proper standardization of protocols, this technology may represent a reliable and effective tool for the analysis of sensitivity in candidate patients to ICI treatment.

### 1.2 FLIM and FRET measurement

**Figure 1:**
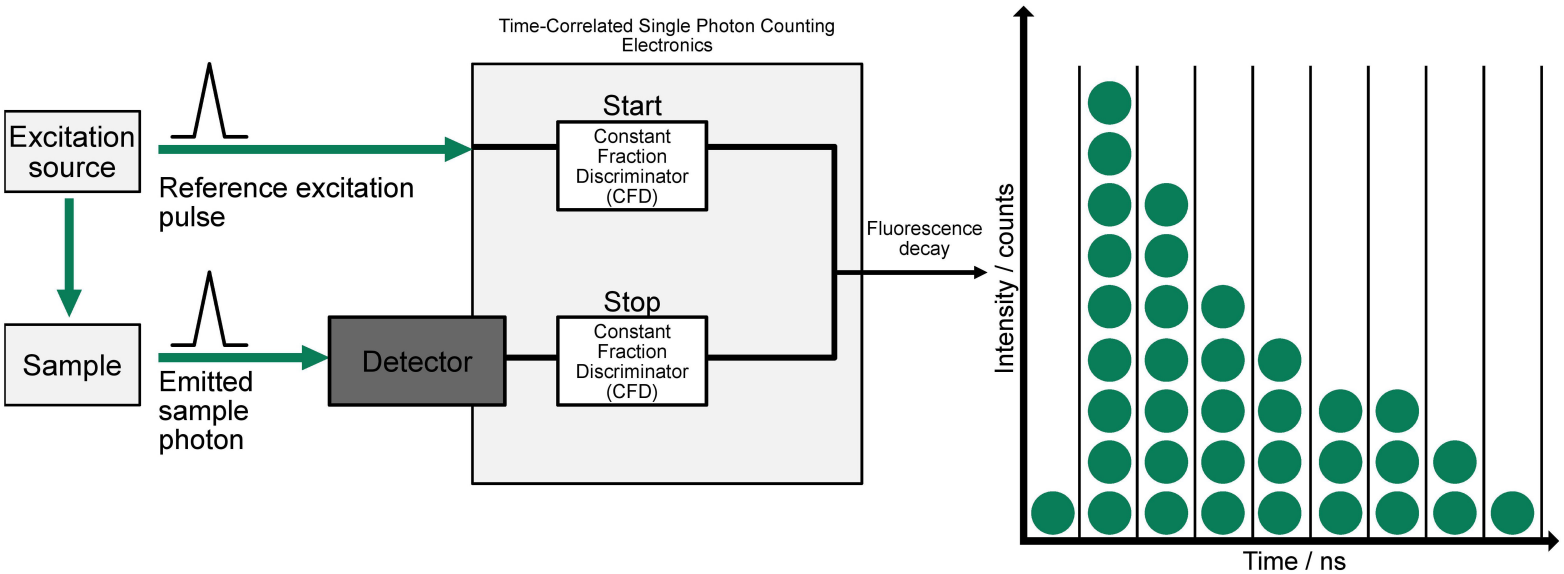
Principles underlying time-domain FLIM. A pulsed excitation source stimulates the sample, and the emitted photon is detected with precise timing relative to the excitation pulse. The system uses two Constant Fraction Discriminators (CFDs) to start and stop the time measurement based on the reference pulse and emitted photon, respectively. This process is repeated to build a histogram of photon arrival times, producing a decay curve that reflects the fluorescence lifetime distribution, shown on the right (adapted from (4)).

**Figure 2:**
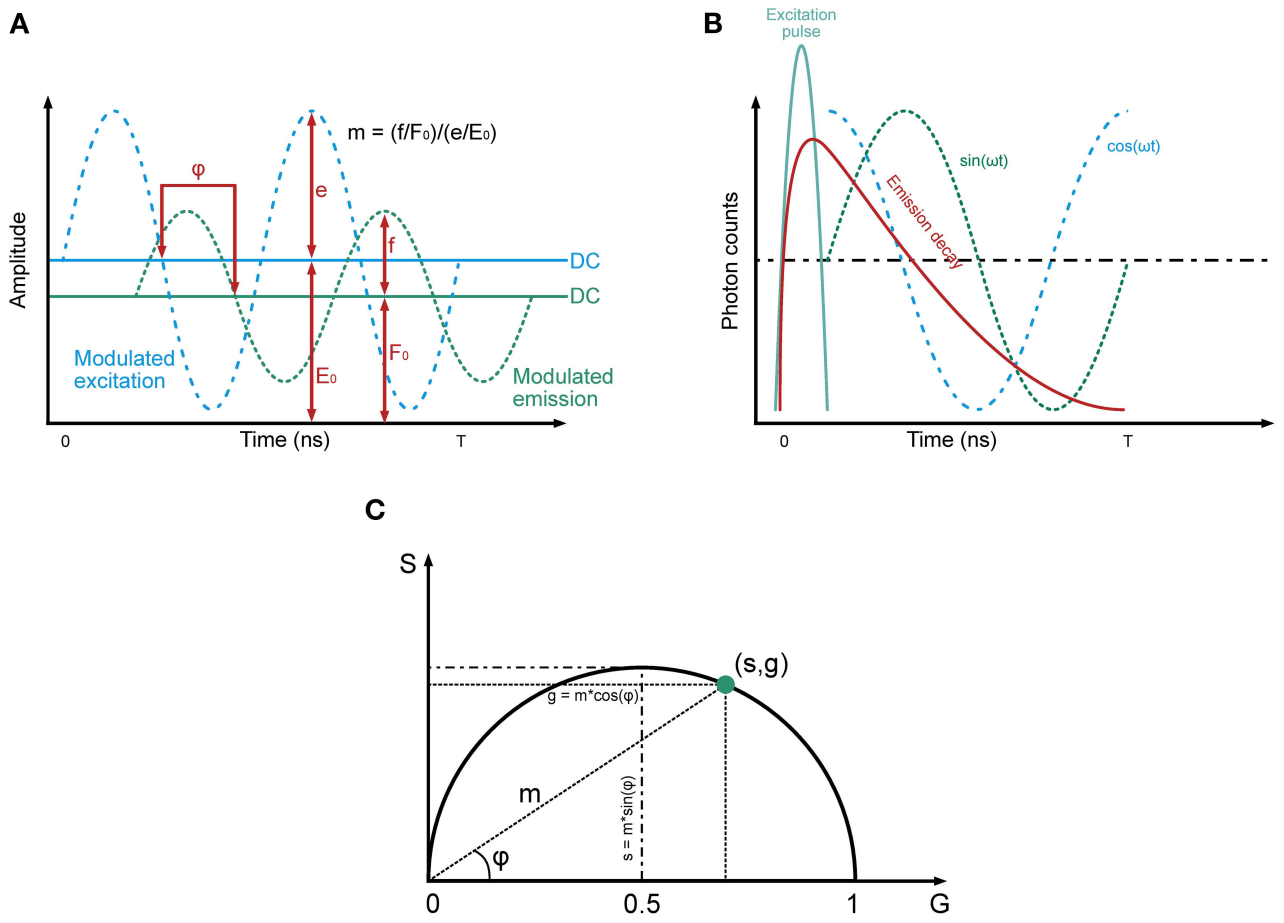
Principles underlying frequency-domain FLIM. The tree panel gives an overview of the principles underlying Frequency-Domain FLIM, highlighting key components of the method. **(A)** Modulated excitation and emission signals: This panel illustrates the sinusoidal modulation of the excitation signal (blue dashed line) and the corresponding modulated fluorescence emission signal (green dashed line). The emission signal exhibits a phase delay (φ) relative to the excitation and a reduction in amplitude, reflecting the properties of the fluorophore. The DC components of the excitation and emission signals (E0 and F0, respectively) are shown as horizontal lines, while the amplitudes (e and f) represent the oscillatory components. The modulation depth (m) is defined as the ratio of the normalized amplitudes of the emission and excitation signals. These parameters are fundamental for determining fluorescence lifetime. **(B)** Emission decay and harmonic components: note the relationship between the time-resolved fluorescence decay (red curve) and its harmonic representation. The excitation pulse (blue line) initiates the fluorescence response, which decays exponentially over time. Superimposed sinusoidal components, represented as sine (green) and cosine (blue) waveforms, highlight how the modulated emission signal can be decomposed into phase and amplitude components. The phase delay and modulation depth extracted from these signals are directly related to the fluorescence lifetime. **(C)** Polar plot visualizes the relationships between the sine (S) and cosine (G) components of the emission signal in the frequency domain. The modulation depth (m) and phase delay (φ) are depicted geometrically, with g=m·cos(φ) and s=m·sin(φ). The point (s,g) lies on a semicircle, reflecting the harmonic relationship between these parameters. This representation allows fluorescence lifetime to be determined from the distance and angle of the point relative to the origin (recreated from “FLIM Analysis using the Phasor Plots”, by Liao SC, Sun Y, Coskun U (5)).

FLIM and FRET are powerful biomedical imaging and molecular biology techniques. FLIM measures the fluorescence decay rate from excited molecules (**Figures 1**, **2**), providing information on the local biochemical environment. On the other hand, FRET detects energy transfer between two fluorophores in close proximity, allowing for the study of molecular interactions and dynamics.

FLIM-FRET techniques, more thoroughly explained in the **Supplementary Material**, measure the fluorescence lifetime of the donor, avoiding contamination from the acceptor. FRET efficiency is determined by comparing the donor’s fluorescence lifetimes in the presence and absence of FRET. This method allows clear visualization of lifetime decreases in regions where FRET occurs (6, 7). The main advantages of FLIM-based FRET measurements include the ability to distinguish between interacting and non-interacting donor fractions, which is crucial for protein-interaction experiments that often involve a mix of interacting and non-interacting proteins (6, 7).

### 1.3 Research scope and questions

This review will provide a comprehensive analysis of the current state of FLIM technology for the qualitative and quantitative evaluation of ICI response. We will highlight the advantages and limitations of this relatively new technology based on the most relevant studies in the recent literature. Lastly, we will address current challenges and future directions for this technology. The questions that guided this review were: Is FLIM equally as effective at quantifying immune receptor expression as IHC? Can FLIM be used to stratify patients for ICI treatment better than IHC? What are its strengths and limitations?

## 2 Materials and methods

### 2.1 Selection criteria

After formulating the research question and reviewing PRISMA methodologies for systematic reviews, the team agreed on a comprehensive literature identification, screening, and documentation approach. Our research included literature focusing on predicting ICI response through FLIM written in either English or Italian. Eligible studies included retrospective cohort studies, case series, *in vitro* experiments, *in vivo* animal models, and other non-randomized observational studies. Exclusion criteria included conference abstracts, reviews, case studies, and studies lacking relevant conclusions.

### 2.2 Search strategy, data extraction, and analysis

**Figure 3:**
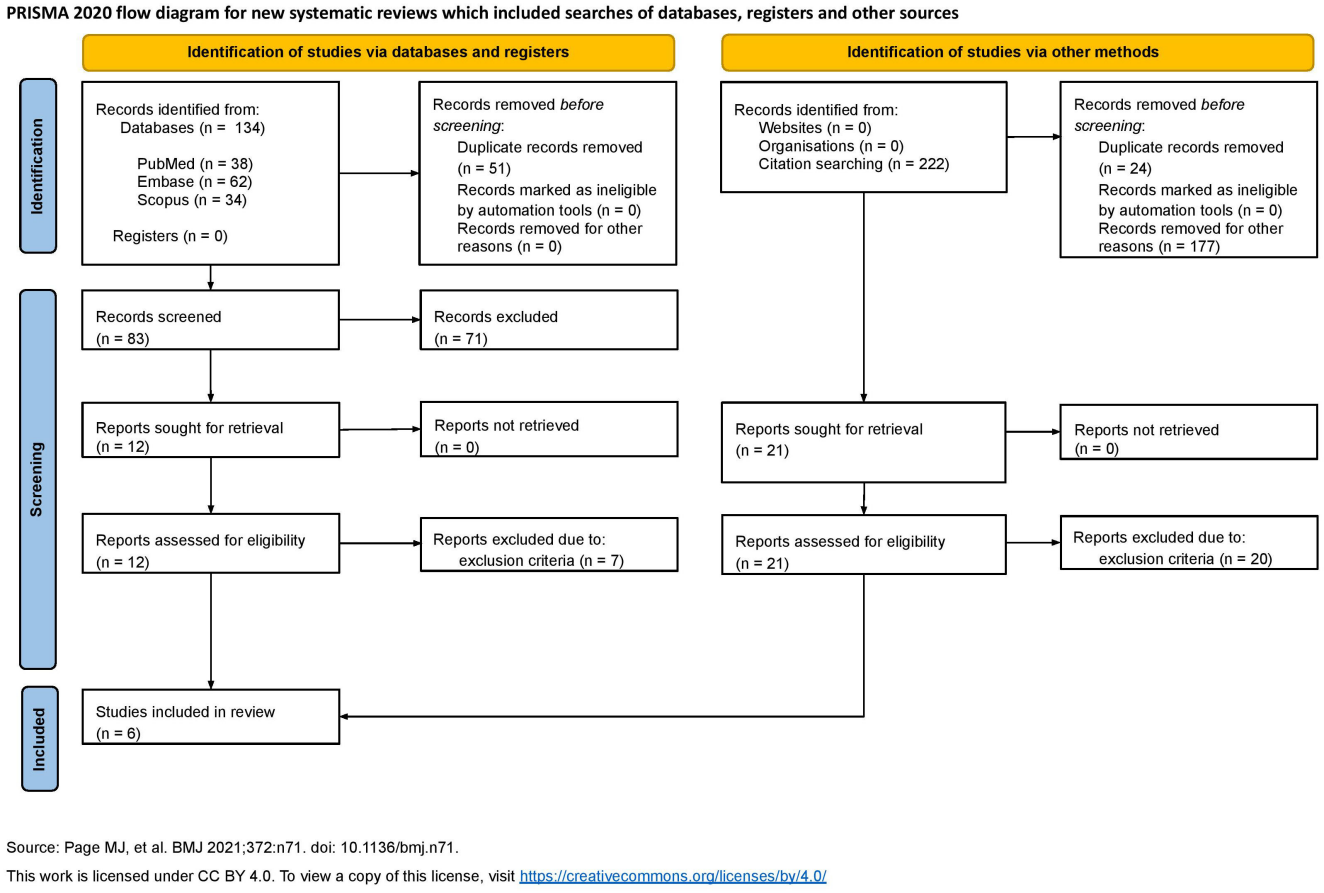
PRISMA 2020 flow diagram.

The PRISMA checklist for systematic reviews was used to draft our work and ensure quality. The search was conducted on April 15, 2025, spanning several sources selected for their relevance to immunology, immunotherapy, and fluorescence imaging. Specifically, the search was performed on PubMed, Embase, and Scopus. Both reference-list scanning and grey literature research were undertaken. Everything is summarized in the flowchart (**Figure 3**).

The following keywords and MeSH were used: “((FLIM) OR (FRET) OR (fluorescence lifetime)) AND ((PD-1) OR (PD-L1) OR (programmed death ligand 1) OR (CTLA4) OR (LAG-3) OR (TIM-3))” on PubMed, “(flim OR fret OR ‘fluorescence lifetime’) AND (‘pd 1’ OR ‘pd l1’ OR ‘programmed death ligand 1’ OR ctla4 OR ‘lag 3’ OR ‘tim 3’)” on Embase, and “(TITLE-ABS-KEY (flim) OR TITLE-ABS-KEY (fret) OR TITLE-ABS-KEY (“fluorescence lifetime”) AND TITLE-ABS-KEY (pd-1) OR TITLE-ABS-KEY (pd-l1) OR TITLE-ABS-KEY (“programmed death ligand 1”) OR TITLE-ABS-KEY (ctla-4) OR TITLE-ABS-KEY (lag-3) OR TITLE-ABS-KEY (tim-3))” on Scopus. The search yielded relevant articles published in “Analytica” by MDPI, “Research Square”, “CTM”, “Cancer Research” by AACR, “Journal of Surgical Oncology” by Wiley, and “Biophysical Chemistry”. Furthermore, reference scanning was performed on these articles. The first- and second-level screening, and data extraction were performed independently by several authors (CC, GF, ML, GM, AM, and FP) to ensure robustness and minimize errors.

### 2.3 Risk of bias assessment

Across the included studies, the overall risk of bias was moderate, **Table 1**. None of the studies were RCTs, and the two human studies (8, 10) were retrospective cohorts with inherent confounding and selection biases. These observational studies did not properly control for all potential prognostic factors, which could significantly influence the observed association between FLIM-based biomarkers and ICI outcomes. However, objective endpoints have been applied (e.g., survival) and, in one case, a blinded multi-site assay analysis to mitigate measurement bias (Sánchez-Magraner et al., 2023). The *in vitro* proof-of-concept study was at low risk of bias, benefiting from a controlled experimental setup and objective readouts. The preclinical animal studies were generally well-conducted but still exhibited some risk of bias common to exploratory animal experiments, such as unclear blinding of outcome assessment and, in one case, non-random group assignment. One murine study did implement randomization for therapy vs. control groups, strengthening its internal validity. Overall, while all included studies had methodological limitations (e.g., retrospective design, small sample sizes, or incomplete reporting of blinding), no study was found to have a high or critical risk of bias. This suggests that the current evidence, though preliminary, is not compromised by fatal bias; still, the moderate risk-of-bias across studies underscores the need for cautious interpretation of the findings and highlights the importance of more rigorous future research (e.g., prospective trials) to confirm FLIM’s predictive value.

**Table 1:** Risk of bias assessment.

Reference	Study type	Risk of bias tool	Bias domains assessed	Overall risk of bias
(8)	Retrospective human biomarker study (multicenter observational)	ROBINS-I (non-randomized studies)	Confounding (no randomization), selection bias (retrospective cohort), outcome measurement (blinding not reported), missing data, selective reporting	Moderate – Retrospective design and single-region sampling may introduce selection bias and confounding, though outcomes (survival) were objective.
(9)	Experimental *in vitro* study (cell membrane assay)	No standard tool (custom domains)	Sample selection and reproducibility, performance consistency (controls with/without drug), detection bias (instrument measurement), reporting transparency	Low – Well-controlled proof-of-concept experiment with appropriate controls (± pembrolizumab) and objective measurements, yielding minimal risk of bias.
(10)	Retrospective human biomarker study (NSCLC patients)	ROBINS-I (non-randomized studies)	Confounding (retrospective with varying patient factors), selection bias (multisite sample selection), performance bias (not applicable – single-arm), detection bias (blinded PD-1/PD-L1 assay), missing data, selective reporting	Moderate – No randomization and potential confounders (e.g., heterogeneity in clinical data) limit causal inference, but blinded quantitative imaging and objective outcomes (survival) strengthen internal validity.
(11)	Experimental *in vivo* study in mice (PD-L1 heterogeneity imaging)	SYRCLE tool (animal studies)	Selection bias (no treatment groups; small sample of tumor-bearing mice), performance bias (not reported if outcome assessment was blinded), detection bias (objective FLIM measurement of PD-L1), attrition (complete data), reporting bias (all outcomes reported)	Moderate – Methodology was exploratory with a small sample (7 TNBC, 4 HCC mice). No intervention was tested, but lack of blinding/reporting details warrants caution. No obvious bias in measurement was noted, though the small scale and unclear randomization procedures yield some uncertainty.
(12)	Experimental *in vivo* study in mice (anti-PD-1 therapy efficacy)	SYRCLE tool (animal studies)	Selection bias (random group allocation to anti-PD-1 vs control), performance bias (likely not blinded to treatment), detection bias (outcome measures: FLIM and tumor response – objective but blinding not stated), attrition (no missing animals), reporting bias (complete outcome reporting)	Low–Moderate – A well-designed preclinical study with randomization to treatment vs control groups and objective imaging outcomes. Some risk remains due to absent mention of blinding and the inherent limitations of an animal model, but overall bias is limited.
(13)	Experimental *in vivo* study in mice (anti-CTLA-4 therapy)	SYRCLE tool (animal studies)	Selection bias (treatment vs control group allocation not fully described), performance bias (blinding of investigators not reported), detection bias (FLIM metabolic readout and response evaluation, likely objective but unblinded), attrition (complete data from 43 mice), reporting bias (full outcome reporting)	Moderate – This animal study tested FLIM on T-cell metabolism as a predictor of anti-CTLA-4 response. It used a reasonable sample size (43 mice) and validated findings with flow cytometry. While no critical flaws were evident, the lack of explicit blinding and potential uncontrolled differences between experimental groups warrant a moderate risk-of-bias rating.

## 3 Results

Our results are summarized in **Table 2**. Among the six articles matching our query, five (8–12) are published studies and one (13) is a preprint. Of these, five (8–11, 13) focus on in-depth analyses of new methods for assessing anti-PD1/PD-L1 therapy response to improve patient stratification, while one (12) explores anti-CTLA-4 immunotherapy by evaluating increased free NADH in tissue samples using FLIM. Three articles investigating PD-1/PD-L1 (8–10) examine the feasibility and correlation between PD-1/PD-L1 interaction (detected by iFRET in *ex vivo* samples) and patients’ responses to immunotherapy; while the other two (11, 13) analyze the concordance of FLIM in detecting PD-L1 expression levels in *in vivo* mouse samples and compare these findings with the IHC PD-L1 score. It is worth noting that three of the five PD-1/PD-L1 studies (8–10) were conducted by the same research group, and two (11, 13) by another, potentially contributing to the apparent focus on this pathway. While our dataset is limited, this distribution may still mirror broader trends in the field, with CTLA-4 representing a less-examined but promising direction for future research.

**Table 2:** Data collected from reference papers regarding FLIM and ICI.

Reference	Target receptor	Study type	Methods	Sample type and size	Key findings	Limitations	Conclusions
(8)	PD-1/PD-L1 and CTLA-4/CD80	Retrospective observational biomarker study	FLIM/iFRET for the quantification of PD-1/PD-L1 and CTLA-4/CD80 interaction in FFPE tumor samples (ccRCC, melanoma, NSCLC), validated with cell assays.	22 patients with ccRCC; 176 patients with melanoma; 60 patients with NSCLC(40 with clinical outcome data).	PD-1/PD-L1 interaction varies, not linked to PD-L1 levels; high interaction correlated to better survival in NSCLC/melanoma; iFRET detects even in PD-L1–negative.	Retrospective design, lack of dynamic post-treatment assessment of interactions, and analysis limited to a single tumor region, which may not capture the full extent of intratumoral heterogeneity.	iFRET predicts outcomes better than PD-L1. High interaction indicates better response to PD-1 blockade. iFRET may improve patient stratification.
(9)	PD-1/PD-L1	Experimental *in vitro* study	FLIM/iFRET assay to provide a quantitative readout of PD-1/PD-L1 interactive states between cell membranes.	80 samples used for CMMA construction (*HT144* cell line, human melanoma samples, and rat brain cortex tissue)	PD-1/PD-L1 binding between immune and melanoma membranes was successfully quantified using membrane microarrays combined with TR-FRET.	Proof of concept of CMMAs feasibility as a tool.	iFRET with membrane microarrays detects PD-1/PD-L1 without live cells, validated with pembrolizumab. The assay is reproducible, low-labor, and adaptable for immune monitoring and personalized therapy.
(10)	PD-1/PD-L1	Retrospective observational biomarker study	Automated, high-throughput FLIM/iFRET assay for the quantification of PD-1/PD-L1 interaction, validating the predictive value for stratification and prognosis with the gold standard.	188 patients with NSCLC	PD-L1 score is less efficient than PD-1/PD-L1 interaction state, measured through FRET efficiency values, in predicting overall survival and therapy response in patients with NSCLC	Retrospective design, high variability in FRET data, incomplete clinical data, and inability to separate tumor-immune from immune-immune interactions.	Measuring PD-1/PD-L1 interaction may be more useful than PD-L1 score by IHC. Prospective studies are needed for validation.
(11)	PD-L1	Experimental *in vivo* imaging in murine models	*In vitro* and *in vivo* viability proof of Fluorescence Lifetimes (FLT) as a way to quantify PD-L1 expression in TNBC or HCC.	30 Eight-week-old C57Bl/6 mice, 23 f with TNBC, 4 m with HCC, 3 f for control	FLIM enables noninvasive, real-time measurement of PD-L1 expression and heterogeneity in tumors, offering a dynamic tool to monitor immunotherapy response beyond traditional biopsies.	Only applicable to superficial tissue due to FLT field depth capabilities.	FLT is a viable detection method for PD-L1 expression.
(12)	CTLA-4	Experimental *in vivo* imaging in murine models	TD-FLIM of NAD(P)H autofluorescence in lymph node T cells for the evaluation of therapy effectiveness, validated through flow cytometry.	43 (C57Bl/6 FoxP3-EGFP) mice with B16F0 melanoma.	FLIM detects T-cell metabolic shift with more free NADH linked to better anti-CTLA-4 response. Responders show higher IFN-gamma and activation markers.	Animal model; clinical applicability to humans requires further investigation.	FLIM of NAD(P)H autofluorescence in lymph node T cells is a potential biomarker for early assessment of anti-CTLA-4 immunotherapy effectiveness.
(13)	PD-L1	Experimental *in vivo* imaging in murine models	TD-FLIM for the detection of intra-tumor PD-L1 heterogeneity using radiolabeled *αPDL1–800* antibodies.	7 mice with triple-negative breast cancer (TNBC) tumors; 4 mice with hepatocellular carcinoma (HCC) tumors	TD-FLIM was able to quantify intra-tumoral heterogeneity of PD-L1, distinguishing between tumor specific and non-specific signals	Needs further studies with larger sample sizes to support clinical translation to humans	TD imaging provides a quantitative measure of PD-L1 expression, useful for assessing tumor heterogeneity and monitoring response to immunotherapy.

Due to substantial heterogeneity across the included studies, particularly in terms of sample type (human tissue, cell lines, and animal models), cancer subtype, and sample size, a quantitative synthesis was not feasible. Instead, a narrative synthesis was conducted to summarize and contextualize the findings.

Sánchez-Magraner et al. (2020) (11) investigated the limitations of the IHC-measured PD-L1 score (currently the gold standard) in predicting immunotherapy response in cancer patients and proposed an alternative approach based on measuring functional PD-1/PD-L1 interactions through iFRET for improved prognostic and predictive value. The study analyzed patients with non-small cell lung cancer (NSCLC), malignant melanoma, and clear cell renal cell carcinoma (ccRCC). Using FLIM combined with amplified signal detection, the authors measured FRET efficiency between PD-1 and PD-L1 molecules across patient samples. These interaction scores were then correlated with PD-L1 scores and with patient survival outcomes. The study included a retrospective cohort of anti-PD-1-treated metastatic NSCLC patients. The results revealed that not only does FRET efficiency vary significantly both between and within tumors, it also has no correlation with PD-L1 score. Notably, patients with higher PD-1/PD-L1 interaction levels exhibited better responses to immunotherapy and improved survival in melanoma and NSCLC cohorts. This suggests that tumors heavily reliant on PD-1-mediated immune evasion are more vulnerable to PD-1/PD-L1 blockade, a finding that undermines the utility of PD-L1 expression as a predictive biomarker. While the study provided compelling evidence that iFRET can capture clinically relevant checkpoint activity, certain limitations remained underexplored. For instance, the study focused on ranking patients based on iFRET efficiency and correlating it with survival time. While it was found to be statistically significant, it doesn’t discriminate between subgroups, both between tumor stages and the undergone treatment. Furthermore, they performed a single-time-point analysis, which inherently ignores dynamic changes that may occur with treatment.

Sánchez-Magraner et al. (2021) (12) developed an assay consisting of cell membrane microarrays (CMMAs) derived from *HT144* cell lines and melanoma samples to assess PD-1/PD-L1 interactions quantitatively. The study involved incubating the CMMAs with cell membranes isolated from peripheral blood mononuclear cells, which expressed PD-1. The PD-1/PD-L1 interaction was then quantified through time-resolved FRET. To validate the specificity of this interaction, they performed the assays both in the presence and absence of Pembrolizumab, an anti-PD-1 ICI. The results showed the assay’s capability to effectively quantify PD-1/PD-L1 interactions. Notably, the interaction was disrupted when pembrolizumab was present, confirming also the assay’s sensitivity to targeted inhibition of PD-1/PD-L1 binding. However, this study represents only a proof of concept of the feasibility of CMMAs as a tool.

Sánchez-Magraner et al. in 2023 (13) expanded on the findings of the 2020 study (11). Their research explored whether quantifying PD-1/PD-L1 interactions in Formalin-Fixed Paraffin-Embedded tissue samples, taken from a cohort of 188 patients with *in-situ* or metastatic NSCLC treated with immune checkpoint inhibitors, could support effective patient stratification and identify candidates for immune checkpoint blockade therapy. This analysis was conducted using a high-throughput, automated, quantitative imaging platform called “QF-Pro”, based on iFRET. The results revealed no correlation between PD-1/PD-L1 interaction and PD-L1 score. The PD-L1 score was found to have a very weak correlation with patient prognosis. Contrarily, PD-1/PD-L1 interaction was shown to have a strong positive correlation (p<0.0001) with the anti-PD-1/PD-L1 therapy response (patients exhibiting high FRET efficiency demonstrated improved survival outcomes). While the study highlights the potential of QF-Pro to quantify PD-1/PD-L1 complex formation and aid in patient stratification, it also presents several limitations. Firstly, the method introduces considerable intra- and inter-patient variability, requiring careful sampling to ensure consistency. The retrospective design limits the ability to confirm predictive value, emphasizing the need for prospective validation. The lack of detailed clinical data, such as smoking status, and the inability to distinguish between tumor-immune and immune-immune interactions, further constrain the findings.

Pal et al. (2023) (13) investigated the use of time-domain (TD) fluorescence imaging to measure the expression of PD-L1 in tumors. Researchers employed a PD-L1-specific antibody labeled with IRDye 800CW (*αPDL1-800*) to perform *in vivo* TD fluorescence imaging in murine models. They conducted both wide-field imaging for superficial triple-negative breast cancer (TNBC) tumors and tomographic imaging for deeper-seated hepatocellular carcinoma (HCC) tumors. The fluorescence lifetime (FLT) of *αPDL1–800* served as a quantitative measure of PD-L1 expression. The study demonstrated that FLT measurements could effectively differentiate between specific and nonspecific accumulation of *αPDL1-800*, allowing for accurate quantification of PD-L1 expression. In TNBC models, FLT imaging revealed significant inter-tumoral heterogeneity in PD-L1 levels. Furthermore, *in vivo* FLT findings correlated well with *ex vivo* assessments, including western blot and immunohistochemistry. In HCC models, TD tomographic imaging successfully quantified PD-L1 expression in tumors located more than 5 mm beneath the surface, highlighting the technique’s capability to assess deep-seated tumors. The research suggests that TD fluorescence imaging offers a robust, non-invasive method for quantifying PD-L1 expression in both superficial and deep tumors. This approach could enhance the assessment of tumor heterogeneity and improve monitoring of responses to immunotherapy, thereby aiding in the selection of appropriate patients for such treatments. The main drawback of this study is the small sample size. Further studies are needed to assess clinical feasibility on humans.

Pal et al. (2025) (11) proved the applicability of time-domain FLIM for noninvasive, quantitative *in vivo* assessment of PD-L1 expression and intertumoral heterogeneity in intact tumor models. Recognizing the limitations of IHC, the authors addressed the inadequacy of *ex vivo*, static, and regionally limited measurements in capturing the dynamic and heterogeneous nature of PD-L1 within and across tumors. The researchers conjugated a monoclonal anti-PD-L1 antibody (clone 29E.2A3) to the near-infrared fluorophore *αPDL1–800* and validated its PD-L1 specificity using both *in vitro* and *in vivo* models. *In vitro*, FLT increased upon binding of *αPDL1–800* to PD-L1, distinguishing it from nonspecifically accumulated probes. This FLT shift correlated linearly with PD-L1 expression levels modulated by IFNγ treatment in *E0771* and *RIL-175* cell lines. Both FLIM microscopy and Western blot analysis (r² = 0.89) were used to strengthen the results. For *in vivo* validation, wide-field TD-FLT imaging was conducted on murine models of triple-negative breast cancer (TNBC, E0771) and hepatocellular carcinoma (RIL-175). The tumor-associated FLT of PD-L1-bound *αPDL1–800* was consistently longer than that of unbound probes in normal tissue, enabling the separation of specific from nonspecific signals. This separation facilitated the calculation of normalized amplitude ratios, which exhibited a robust correlation with PD-L1 expression measured by Western blot (r² = 0.96), outperforming fluorescence intensity alone. The technique was further applied to monitor immunotherapy-induced PD-L1 upregulation in anti-PD-1 treated TNBC mice, where both FLT and aT/aNS ratios detected significant increases in PD-L1 expression relative to controls (p<0.01). Importantly, the study demonstrated FLT imaging capacity for quantifying baseline heterogeneity and treatment-induced modulation of PD-L1 in superficial and deep-seated tumors via planar and tomographic imaging. While this study demonstrated the applicability *in vivo* on mice, translation into human practice is hindered by the field depth of FLT, which doesn’t allow for measuring expression in non-superficial tissues.

Isozimova et al. (2023) (12) aimed to validate the use of NAD(P)H autofluorescence lifetime of T cells within lymph nodes as a predictive biomarker for response to anti-CTLA-4 immunotherapy. The research focused on assessing metabolic changes in immune cells as indicators of treatment efficacy. The study utilized C57Bl/6 FoxP3-EGFP transgenic mice with B16F0 melanoma implanted near the inguinal lymph node. Mice were treated with anti-CTLA-4 antibodies. Lymph nodes were harvested 1–2 days post-treatment and analyzed with a FLIM-equipped microscope. Decay curves were fitted into a model to determine NAD(P)H lifetime components. Flow cytometry assessed activation markers (CD25, CD69) and cytokine production (IFN-γ) in CD4+ and CD8+ T cells. Anti-CTLA-4 treatment led to a trend towards reduced tumor growth compared to controls, with significant differences observed on day 11. However, variability in tumor response was noted, with some mice showing pronounced growth inhibition and others minimal response. FLIM data revealed that responder mice exhibited a higher proportion of the free NADH form associated with glycolysis than non-responders. This shift suggests enhanced metabolic activity in activated T cells. The average NAD(P)H lifetime did not differ significantly between groups. Responder mice showed increased expression of activation markers CD25 and CD69, and higher IFN-γ production in both CD4+ and CD8+ T cells, indicating effective immune activation. Non-responders did not exhibit these changes, aligning with FLIM findings. One key limitation of this study is that it did not explore the long-term effects of immunotherapy or the correlation between early metabolic changes and long-term treatment outcomes. Furthermore, the cohort of mice was limited in size, requiring further studies for human applicability.

## 4 Discussion

### 4.1 IHC as the gold standard

Currently, the standardized FDA-approved method in almost all Pathology Departments all over the globe to quantify IC expression (i.e., PD-L1) is immunohistochemistry, through approved kits with specific antibodies (i.e., 28-8, 22C3, SP263, and SP142) (14). Based on immunohistochemical analysis, compared to non-expressing subjects, patients with immune checkpoint overexpression present with a stronger antitumor activity and are more likely to benefit from ICI (13, 15, 16). However, although it is an “easy-to-use”, fast, and inexpensive method, as for other immunohistochemical evaluations, it can only provide a momentary picture of the microenvironmental status in a confined region of *ex vivo* specimen; furthermore, protein expression could be influenced by the concentration of fixative used or other variables related to instruments used, or the inter-observer variability on data interpretation (17, 18).

### 4.2 FLIM

FLIM has evolved exponentially from 1988, when it was first introduced, until today; merging theoretical techniques with biomedical research. Nowadays, it can be used in combination with other imaging technologies to gather further information about the cellular microenvironment.

Recently, FLIM has gained technological advancements resulting in an improvement in the precision of analysis, as well as a broadening of this technology’s applications. These advancements have allowed it to match the precision of IHC for the analysis of ICI therapy at a microenvironmental level (8–13). However, it is unclear how responders can be differentiated from non-responders. Traditionally, IHC has been used to quantify the expression of receptors, but the expression alone does not greatly correlate to efficacy. On the contrary, functional engagement measured with FLIM between drug and receptor is a promising predictor for the success of the therapy by measuring it independently from their concentration. The clinical application would mean the inclusion of low-expressing patients who would normally be excluded from the therapy or the exclusion of non-responding high-presenting patients who would needlessly suffer the side effects.

### 4.3 FLIM’s advantages & limitations

FLIM and FRET are reliable imaging techniques that could overcome some of the limitations of IHC. **Table 3** shows a direct comparison between the technical and practical capabilities of IHC and FLIM. While IHC is the gold standard for predicting the body’s response to various ICIs, its limitations have become increasingly evident (2). Despite the scarce research on this topic, the usage of FLIM and FRET to test ICI efficacy have shown promising results by providing real-time, non-invasive insights into molecular interactions such as PD-1/PD-L1 engagement, surpassing the static and limited biopsy samples used in IHC. FLIM detected treatment-induced changes in tumors *in vivo* just 2 days post-treatment, which is earlier than detectable changes in tumor volume (31). Furthermore, FLIM-FRET allows for the visualization of checkpoint interactions at a microscopic level, providing crucial information about the functional state of these molecules as shown by iFRET which detected significant interaction states in patients who were PD-L1 negative according to IHC (8). FLIM can help detect cells’ *in vivo* metabolism with no phototoxicity and in real-time. FLIM’s capability to quantify functional interactions offers a more comprehensive approach, potentially improving the stratification of patients for immunotherapy and reducing the ambiguity associated with IHC-based assays. It is important to underline that FLIM finds its greatest potential *in vivo* and relies on the fact that its results, unlike traditional fluorescence microscopies, are not dependent on the change in fluorescence intensity but on the lifetime. FLIM, with or without FRET, still suffers from major drawbacks that vary depending on its specific application. The use of FLIM technology in the analysis of *ex vivo* samples proves it is non-superior to more widespread methods since metabolic microenvironment characteristics are lost in the transition from the *in vivo* to the *ex vivo*, limiting its ability to provide accurate insights into molecular interactions.

**Table 3:** Comparison between FLIM and IHC.

	FLIM	IHC	References
Principle	Fluorescence lifetime measurement	Detection of antigen–antibody binding	(3, 19–22)
Contrast Mechanism	Differences in fluorescence lifetime	Chromogenic/fluorescent signal	(3, 19–22)
Multiplexing	High (lifetime-based separation)	Moderate (spectral separation)	(1, 22–24)
Functional Data	Yes (biochemical and metabolic information)	Limited (depends on the marker)	(2, 21, 22, 25)
Sample Type	Live or fixed samples; 2D or 3D	Fixed or fresh samples; 2D	(20–22, 26–28)
Clinical Use	Research and emerging clinical applications	Routine clinical pathology	(20, 22, 28)
Time	Depending on the complexity, estimation model, and multiplexing, up to multiple days.	Hours up to a day	(2, 7, 23, 25, 29)
Training required	A good understanding of fluorescence theory, FLIM system principles, and experimental procedures is required due to the highly specialized equipment	Standard technician or pathologist training, with high degrees of automation	(7, 23, 25, 29)
Cost	High, due to advanced technological equipment. Some setups (e.g., frequency-domain FLIM) are relatively less expensive	Low, as the reagents constitute the main cost	(3, 7, 24, 26, 29, 30)
Time to detect changes after treatment	As early as 2 days post-treatment (via metabolic contrast)	At least 6 days post-treatment (via changes in tumor volume)	(7)

Hence, the true power of FLIM resides in its *in vivo* application. One viable way to adopt FLIM in an *ex vivo* context would be through FLIM used with Raman spectroscopy (32) as the tissue cryosections partially maintain the *in vivo* microenvironment. However, FLIM-FRET detection probes are unsuitable when using this technique due to the need for a non-frozen tissue for their employment rendering this technique limited. The probes are a major issue even in the *in vivo* applications of FLIM-FRET since they are scarce in number and the existing ones yet unsuitable for human patients.

FLIM also presents some minor challenges that further research can work to resolve. For example, since the lifetime duration computation is based on statistics, the higher the number of iterations, the higher the precision and certainty of measurements. This implies that a drastic increase in time is required to obtain robust values, especially in multiplexing applications (33). Notably, scalability for clinical use is being addressed by innovations such as GPU-accelerated high-speed FLIM, which significantly reduces imaging and processing times (25). Deep learning approaches, such as Phasor U-Net, automate and accelerate lifetime extraction and multiplexing, minimizing manual intervention and enabling rapid, accurate analysis even with limited photon counts (34). High-throughput acquisition systems using array detectors and parallelized photon counting further increase scalability (35).

Another problem of FLIM *in vivo* applicability is the depth of measurements (31). Until 2024, the number of tumors analyzed *in vivo* with this technique is minimal (mainly melanomas due to their easily accessible location). Tissue depth penetration is limited as with all optical imaging modalities, and with two-photon FLIM it is around 100-130 μm (36), *in vivo* visualization of deep tissues (i.e., intestine, kidneys, liver) is currently limited to invasive approaches.

Since FLIM-FRET works with standard confocal microscopy, the maximum resolution possible is 200–250 nanometers (37), which is enough to make it a valuable option in studying molecular interactions and changes in the microenvironment. However, it is not comparable to other microscopy techniques (i.e., electron microscopy) although they have other serious drawbacks such as phototoxicity.

An additional challenge associated with FLIM-FRET is the complexity and the technical expertise required for its implementation. It requires strict protocols to reduce interference from extrinsic factors (i.e., pH, temperature, *etc.*) that may affect fluorescence decay times (31) and a profound knowledge of the biological environments and pathways involved.

Despite these barriers, commercial development is underway, companies such as JenLab (38–40) are offering FLIM-based devices for dermatological applications, indicating momentum towards clinical implementation.

Also, the cost and resource intensity of FLIM-FRET systems pose a significant barrier; the advanced imaging equipment required is expensive and often requires specialized maintenance. The economic constraint can hinder the broader adoption of FLIM-FRET in clinical practice, despite its potential benefits.

### 4.4 Future perspectives

The utilization of FLIM and FLIM/FRET is expected to have a great impact on future clinical practice (41, 42) considering the effect on cancer patients’ diagnosis given the high specificity and sensitivity of the technique, particularly regarding genetically encoded biosensors reviewed by Vu et al. (41). In 2023 it has been shown that high precision and accuracy (respectively closeness of known values among them and closeness of known values with the true one) guarantee a delved and highly specific landscape of the cellular metabolism and molecular interactions previously presented in the ICI section.

This novel technology encompasses the current trend of personalized precision medicine. We are gradually diving into having a treatment specific to each patient for high-quality care and patient management.

The current state-of-art of FLIM technology strongly suggests that intraoperative guidance use of FLIM has been emerging as a relevant and consistent future application of the mentioned technology. In this setting, FLIM is invasive, as it requires direct access to tissue during surgical procedures, but it enables real-time imaging capabilities (42). At the same time, non-invasive applications are also advancing, particularly in dermatology (38–40). These highlight that there is still an optimal margin to further enhance this microscopy in both domains.

Practical pipeline development for broader clinical integration of FLIM involves creating comprehensive training programs for laboratory technologists and pathologists, deploying automated, ready-to-use FLIM systems with standardized protocols, and embedding FLIM modules into existing histopathology and cytometry platforms. Open-source toolkits like FLIMJ facilitate integration with established image analysis workflows, reducing the barrier for adoption in clinical laboratories (43). Regulatory pathway development and multi-institutional validation studies are essential for clinical acceptance.

Overall, the topic remains mostly underexplored, and further research is needed to better understand the potential of known and alternative immune checkpoint pathways such as CTLA-4, TIM-3, and LAG-3.

## 5 Limitations

Although our article is based on robust guidelines for drafting, it does have some limitations. The exclusion of articles written in languages other than English and Italian may have limited the scope of our literature search. Restricting our search to only PubMed, Scopus, and Embase may have excluded relevant studies available in other databases, journals, or websites. Additionally, the keywords and MeSH employed for the research may have excluded other relevant studies.

## 6 Conclusions

The quantification of the Immune Checkpoint Inhibitor response remains a critical challenge in cancer therapy. Traditional techniques like immunohistochemistry do not have high accuracy and commonly fail to meet the desired reliability in predicting response. FLIM and FRET offer a promising alternative by enabling real-time visualization and quantifying molecular interactions within the tumor microenvironment.

Other novel imaging techniques, integrating emerging platforms like CyTOF, multiplexed immunofluorescence, and spatial proteomics, should be further investigated to unlock new avenues for biomarker discovery and therapeutic stratification.

Future research should focus on refining FLIM and FRET methodologies to quantify the effects of Immune Checkpoint Inhibitors, linking them to patient outcomes. This could be explored through the use of current biomarkers, novel biomarkers, and innovative FLIM and FRET protocols. By combining these advanced approaches, there is the potential to make a breakthrough in the cancer immunotherapy landscape, aiming at a more personalized treatment for patients.

## Data Availability

The original contributions presented in the study are included in the article/**Supplementary Material**, further inquiries can be directed to the corresponding author/s.
