# Supplementary material for: Predicting immune checkpoint inhibitors response via fluorescence lifetime imaging microscopy: a systematic review
Source: Front Immunol. 2025 Oct 6;16:1626608. doi: 10.3389/fimmu.2025.1626608 (PMC12535979; doi:10.3389/fimmu.2025.1626608)
Supplement: Supplementary file 1 [file Presentation1.pdf]

PD-1/PD-L1

The connection between the transmembrane protein known as Programmed Cell Death Ligand (PD-L1) and PD1 on lymphocytes infiltrating the tumor environment indicates that evading the system is a frequent result of producing immunomodulatory ligands in cancerous growths. Two research papers (1-2) have emphasized the significance of the PD1L/PD-L1 pathway as a checkpoint targeted in various cancer types, like lung carcinoma and melanoma, among others (1-2). Its primary function involves suppressing responses and promoting self-tolerance by altering T cell behavior and inducing T cell death while inhibiting regulatory T cell demise. PD-L1 is commonly believed to act as a brake on the system's response to cancer cells by limiting the system's reaction to them when it combines with PD-L1. The interaction between PD-1 and PD-L1 stops PD-1-positive cells from releasing signaling proteins, induces programmed cell death, and targets specific white blood cells. This highlights the role of the PD-1/PD-L1 axis as one of the major targets of cancer therapy. PD1 is a receptor that sits on activated T cells, B cells, and myeloid cells and serves the main purpose of restricting T cell activity in peripheral tissue during inflammation to avoid autoimmunity. PD-L1 meanwhile is fluorescently marked on the surface of tumor cells, antigen cells, and other non-immune cells. After binding PD-L1 on tumor cells or Antigen Presenting Cell, PD-1 on T cells triggers a signal that then recruits and binds phosphatases (SHP-2) to the PD-1 intracellular domain. As a result, the phosphatase removes the phosphate from important signaling molecules in the T cell activation pathway, leading to less T cell signaling and T cell proliferation, and therefore less cytokine production (IL-2 and IFN-γ). This pathway is exploited by tumors as they often overproduce circulating (c)PDL1 which inhibits tumor antigen-specific T lymphocytes. Moreover, PD-1 and PD-L1 interactions can enhance the oncogenic properties of the tumor microenvironment. These drug candidates are

designed to target the interaction, to prevent the inhibitory effect, and to restore T cell functions and other immune cells that become inactive due to cancer cells (3).

Drugs targeting the interaction between PD-1 and PD-L1 can enhance the immune response of the body to cancer cells. These drugs are classified into two groups: PD-1 Inhibitors such as Nivolumab (Opdivo), Pembrolizumab (Keytruda), and PD-L1 Inhibitors like Atezolizumab (Tecentriq), Durvalumab (Imfinzi), Avelumab (Bavencio). PD-1 inhibitors bind to PD-1 while PD-L1 inhibitors block PD-1 and PD-L1 interaction. The competitive blockade of the interaction between PD-1/PD-L1 leads to a stronger immune response against the tumors (4-5).

CTLA-4

Cytotoxic T-lymphocyte-associated protein 4 (CTLA-4) was first identified in 1991 as an alternative receptor for the integral membrane protein B7. It is a surface protein receptor that acts as an immune checkpoint; it downregulates immune responses by suppressing T-cell responses, although its precise mechanism of action is still debated. The protein is composed of three parts: an extracellular V domain, a transmembrane domain, and a cytoplasmic tail. Its clinical significance, outside the research in cancer immunotherapy, is noteworthy, as variants in the gene encoding for CTLA-4 have been associated with a plethora of autoimmune diseases. CTLA-4 and its counterpart, CD28 while being responsible for opposite effects, share the same two ligands, CD80 and CD86. The ligands are different biophysically but share the same effect. They are often referred to as B7 molecules. As CD28 drives the activation of T-cell responses via co-stimulation of T cells, together with TCR, the affinity of CD28 for both CD80 and CD86 is relatively high. CTLA-4, however, binds both ligands with a higher affinity than its counterpart. This is of utmost importance, as had it not been this way, the downregulation of adaptive immunity responses would be impossible.

CTLA-4 is stored in T cells. Its expression is mainly endocytic, being found primarily in intracellular compartments, following clathrin-mediated endocytosis. CTLA-4 is mostly expressed in Treg cells (FoxP3+) and in activated conventional T cells, where most of it (90%) can be found intracellularly. (6)

CTLA-4-expressing Treg cells act in repressing immune response via trans-endocytosis, effectively sequestering CD80/CD86 and rendering the interaction of the ligands with CD28 impossible. The tumor microenvironment can use this ability in its favor, upregulating the expression of CTLA-4-expressing Treg cells to suppress immune responses. It is from these interactions that the possibility of using anti-CTLA-4 drugs to avoid suppression of the

immune response in the cancer microenvironment, led to the development of drugs such as Tremelimumab.

Principles of FLIM

FLIM is a technique that measures the fluorescence lifetime of a fluorophore or autofluorescent component, which is influenced by its molecular environment rather than its concentration. This feature differentiates FLIM from intensity-based measurements, as it does not require special ratiometric fluorophores, allowing for a broader selection of fluorescence markers and access to a wider range of cellular parameters. (7)

Moreover, FLIM is non-invasive and non-destructive, making it suitable for live-cell imaging and real-time analysis of biological processes.

The data collected (i.e. wavelength, experiment time, and the polarisation of the light) is obtained over three spatial dimensions. (7)

The fluorescence decay times of most autofluorescent molecules are influenced by factors such as protein binding, metabolic state, and oxygen concentration. The differences in lifetime are analyzed, providing information about the metabolic state and constitution of the tissue; on the other hand, exogenous fluorophores allow us to explore other environmental characteristics and protein interactions. (7)

When a molecule absorbs a photon, it enters an excited state. After the excitation, it can either return to the ground state by emitting a photon, internally converting the absorbed energy into heat and transferring the heat to the molecular environment, or it can cross into the triplet state and return to the ground state.

The fluorescence decay function is obtained by exciting the fluorophores with short laser pulses and analyzing the decay with high temporal resolution (picoseconds to nanoseconds). (7)

FLIM is employed to differentiate various fractions of the same fluorophore in different states of interaction with its environment. Some applications include ion quenching, oxygen quenching, fluorophore binding, pH sensitivity, FRET, and viscosity measurements. Ion quenching refers to the process where fluorescence or phosphorescence is reduced by the presence of specific ions, such as calcium (Ca^{2+}) and chloride (Cl^{-}), which interact with the fluorophores and alter their excited state. Similarly, oxygen quenching occurs when oxygen molecules diminish fluorescence, particularly in molecules with longer lifetimes. This phenomenon is particularly relevant for endogenous fluorophores like NADH and FAD, where oxygen serves as an indicator of mitochondrial oxygen consumption. These mechanisms are essential for understanding cellular processes. (8-9)

FLIM techniques are divided into time-domain and frequency-domain methods.

Time-Domain FLIM by TCSPC: Time-Correlated Single Photon Counting (TCSPC) involves scanning the sample with a focused high-frequency pulsed laser. It works by exciting a molecule with a photon that returns to the ground state after a specific amount of time. Simultaneously to the emission of the photon, a signal is sent to the detector which prepares to capture the energy released from the decaying molecule. The time between the activation pulse and the decay is collected with the associated spatial coordinates. The recording continues over multiple scan frames to achieve the desired signal-to-noise ratio. TCSPC provides the highest time resolution among FLIM methods. (8,10)

A simplified overview of TCSPC producing a histogram of timed events is summarized in Fig.1.

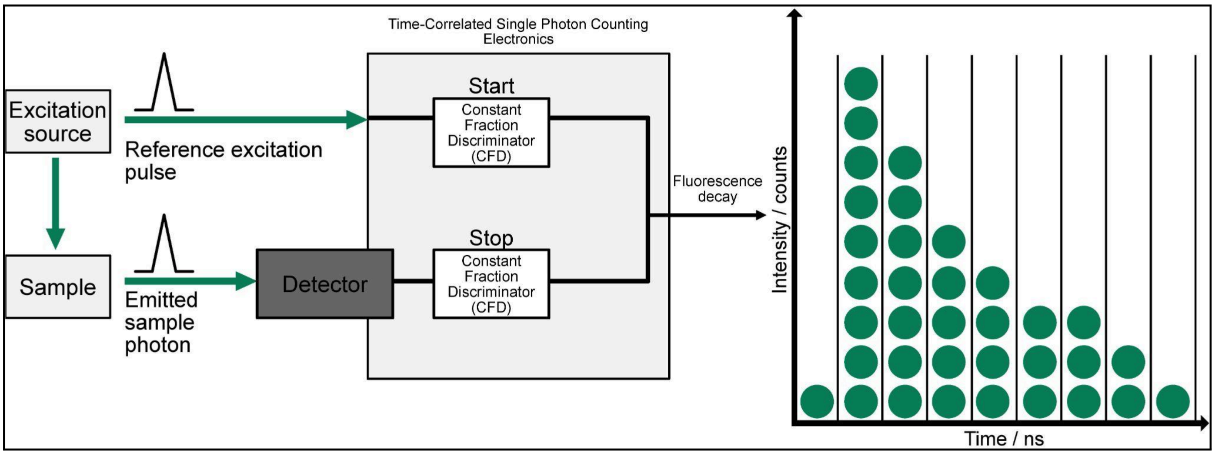

FIGURE 1
Principles underlying time-domain FLIM. A pulsed excitation source stimulates the sample, and the emitted photon is detected with precise timing relative to the excitation pulse. The system uses two Constant Fraction Discriminators (CFDs) to start and stop the time measurement based on the reference pulse and emitted photon, respectively. This process is repeated to build a histogram of photon arrival times, producing a decay curve that reflects the fluorescence lifetime distribution, shown on the right (adapted from (9).).

Finally, we obtain a three-dimensional data array with a time value and spatial coordinates (x,y). TCSPC FLIM benefits from the optical sectioning capability of confocal or multiphoton scanning, capturing data from a well-defined sample plane without out-of-focus

fluorescence contamination. Additional parameters, such as photon wavelength, multiplexed excitation laser wavelengths, or periodic sample stimulation times can be implemented. (7)

Fluorescence decay parameters in time-domain FLIM are extracted through iterative convolution processes, optimizing model parameters for the best fit to photon counts in time channels. The final FLIM image assigns brightness to total photon counts per pixel and color to selected decay parameters, which include single-exponential decay lifetimes, amplitude/intensity-weighted average lifetimes, or lifetime/amplitude ratios. (7)

Frequency-Domain FLIM: In frequency domain, time-domain data (intensity values in subsequent time channels) are processed via Fourier transform into amplitude and phase values in multiples of the signal repetition frequency. This data is directly used to characterize the fluorescence decay behavior of the sample. (8-9)

Frequency-domain FLIM utilizes wide-field excitation with a gain-modulated camera or a scanner with gain-modulated point detectors, of which gain is modulated by an oscillator. The advantage is that the difference frequency is independent from the laser frequency. It is also much lower than the laser frequency. Frequency-domain systems can therefore also be used with high-frequency pulsed lasers. Photon efficiency depends on various instrumental details. (8-9)

Comparisons show that TCSPC FLIM offers better signal-to-noise ratios at low concentrations and count rates, while both techniques converge at high concentrations.

Streak cameras, used with laser scanning microscopes, provide single-photon sensitivity and excellent time resolution but lack depth resolution. (8-9) For a comprehensive overview of the principles underlying Frequency-Domain Fluorescence Lifetime Imaging Microscopy (FD-FLIM), see Fig.2.

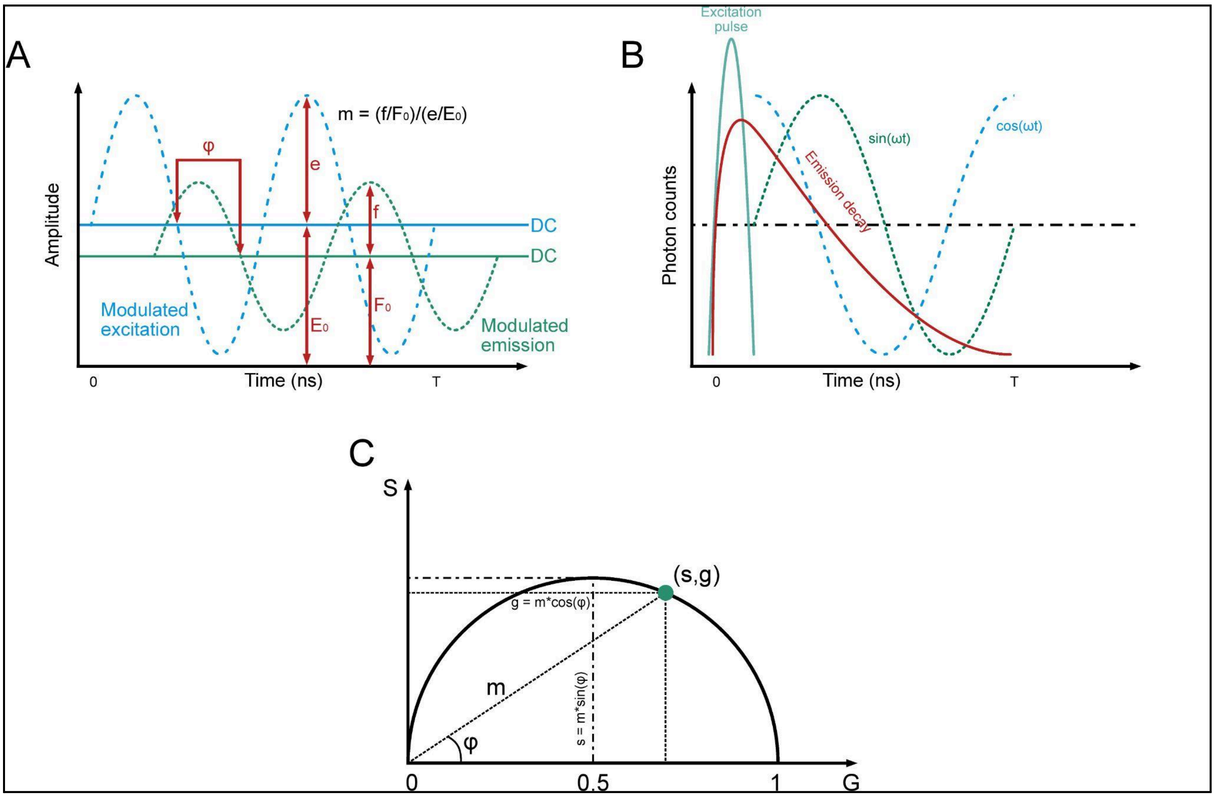

FIGURE 2

Principles underlying frequency-domain FLIM The tree panel gives an overview of the principles underlying Frequency-Domain FLIM, highlighting key components of the method. (A) Modulated excitation and emission signals: This panel illustrates the sinusoidal modulation of the excitation signal (blue dashed line) and the corresponding modulated fluorescence emission signal (green dashed line). The emission signal exhibits a phase delay (j) relative to the excitation and a reduction in amplitude, reflecting the properties of the fluorophore. The DC components of the excitation and emission signals (E0 and F0, respectively) are shown as horizontal lines, while the amplitudes (e and f) represent the oscillatory components. The modulation depth (m) is defined as the ratio of the normalized amplitudes of the emission and excitation signals. These parameters are fundamental for determining fluorescence lifetime. (B) Emission decay and harmonic components: note the relationship between the time-resolved fluorescence decay (red curve) and its harmonic representation. The excitation pulse (blue line) initiates the fluorescence response, which decays exponentially over time. Superimposed sinusoidal components, represented as sine (green) and cosine (blue) waveforms, highlight how the modulated emission signal can be decomposed into phase and amplitude components. The phase delay and modulation depth extracted from these signals are directly related to the fluorescence lifetime. (C) Polar plot visualizes the relationships between the sine (S) and cosine (G) components of the emission signal in the frequency domain. The modulation depth (m) and phase delay (j) are depicted geometrically, with g=m·cos(j) and s=m·sin(j). The point (s,g) lies on a semicircle, reflecting the harmonic relationship between these parameters. This representation allows fluorescence lifetime to be determined from the distance and angle of the point relative to the origin (adapted from (12).).

In frequency-domain FLIM, phase and modulation degree values are obtained per pixel. Phasor analysis defines and displays a pointer (phasor) in a polar plot, using phase as the pointer's angle. Pixels with multiexponential decay profiles appear inside the semicircle of the plot, with different pixel signatures color-coded in the image. This phasor analysis approach can also be applied to Fourier-transformed time-domain data.(7)

Principles of FRET

FRET involves two fluorophore molecules: a donor and an acceptor. Energy is transferred from the donor to the acceptor via a dipole-dipole interaction without the involvement of light emission and absorption. FRET results in quenching of the donor fluorescence and a subsequent decrease in donor lifetime. By labeling different proteins with donor and acceptor fluorophores, FRET can verify physical interactions between proteins and measure distances on the nanometer scale. (7,11)

iFRET is a variant of steady-state FRET that quantifies molecular interactions based on fluorescence intensity measurements of donor and acceptor fluorophores. Therefore, 'FRET efficiency' is derived from the intensity ratio of donor and acceptor fluorescence. However, this method faces challenges such as Donor Bleedthrough (overlap of donor fluorescence into the acceptor emission band), Direct Excitation (contribution from directly excited acceptor molecules), and Labeling Stoichiometry (variations in labeling efficiency). (7,11)

References

1. Salmaninejad A, Valilou SF, Shabgah AG, Aslani S, Alimardani M, Pasdar A, et al. PD-1/PD-L1pathway: Basic biology and role in cancer immunotherapy. JCell Physiol. (2019)234:16824–37. doi:10.1002/jcp.28358
2. Sanguedolce F, Zanelli M. Assessing PD-L1expression in different tumor types. In: Springere Books (2023). p.1–21. doi:10.1007/978-3-030-80962-1_168-1
3. Ishida Y,Agata,Shibahara K,Honjo T. Induced expression of PD-1, a novel member of the immunoglobulin gene superfamily, upon programmed cell death. EMBOJ. (1992)11:3887–95.doi:10.1002/j.1460-2075.1992.tb05481.x
4. Sharma P, Allison JP. Immunecheckpoint targeting in cancer therapy: toward combination strategies with curative potential.Cell. (2015)161:205–14.doi:10.1016/ j.cell.2015.03.030
5. Grossman JE, Vasudevan D, Joyce CE, Hildago M. Is PD-L1 a consistent biomarker for anti-PD-1 therapy? The model of balstilimab in a virally-driven tumor. Oncogene. (2021) 40:1393–5.doi:10.1038/s41388-020-01611-6
6. Pal R, Krishnamoorthy M, Matsui A, et al. Fluorescence lifetime imaging enables in vivo quantification of PD-L1 expression and intertumoral heterogeneity. Cancer Res. (2025) 85:618–32.doi:10.1158/0008-5472.CAN-24-0880
7. Morone D, Autilia FD, Schorn T, Erreni M, Doni A. Evaluation of cell metabolic adaptation in wound and tumour by Fluorescence Lifetime Imaging Microscopy. Sci Rep. (2020) 10. doi:10.1038/s41598-020-63203-4
8. Datta R, Heaster TM, Sharick JT, Gillette AA, Skala MC. Fluorescence lifetime imaging microscopy: fundamentals and advances in instrumentation, analysis, and applications. JBiomedOptics. (2020) 25:1 .doi:10.1117/1.jbo.25.7.071203
9. Datta R, Gillette A, Stefely M, Skala MC. Recent innovations fluorescence lifetime imaging microscopy for biology and medicine. JBiomedOptics. (2021) 26. doi:10.1117/1.jbo.26.7.070603
10. Edinburgh Instruments. What is Time-Correlated Single Photon Counting? Edinburgh Instruments Blog. 28 June 2023. Available from: https://www.edinst.com/blog/what-is-tcspc/ [Accessed 15 April 2025].
11. Pietraszewska-Bogiel A, Gadella TWJ. FRETmicroscopy: from principle to routine technology in cell biology. J Microscopy. (2010) 241:111–8. doi:10.1111/j.1365-2818.2010.03437.x
12. Liao S, Sun Y, Coskun UC. FLIM Analysis using the Phasor Plots. (2015).
